# Adaptation and multicentre validation of a patient-centred outcome scale for people severely ill with COVID (IPOS-COV)

**DOI:** 10.1186/s12955-023-02102-4

**Published:** 2023-03-24

**Authors:** Mevhibe B. Hocaoglu, Fliss E. M. Murtagh, Catherine Walshe, Rachel L. Chambers, Matthew Maddocks, Katherine E. Sleeman, Adejoke O. Oluyase, Lesley Dunleavy, Andy Bradshaw, Sabrina Bajwah, Lorna K. Fraser, Nancy Preston, Irene J. Higginson, the CovPall study team, Andrew Bradshaw

**Affiliations:** 1 Florence Nightingale Faculty of Nursing, Midwifery & Palliative Care, Cicely Saunders Institute of Palliative Care, Policy and Rehabilitation, King’s College London, Bessemer Road, London, SE5 9PJ UK; 2 Wolfson Palliative Care Research Centre, Hull York Medical School, University of Hull, Hull, UK; 3 International Observatory on End-of-Life Care, Division of Health Research, Lancaster University, Lancaster, UK; 4 Health Sciences, University of York, York, North Yorkshire, UK; 5 King’s College Hospital NHS Foundation Trust, Denmark Hill, London, UK; 6 Global Health and Social Medicine, Harvard Medical School, Boston, USA

**Keywords:** Patient-centred outcomes, Severe COVID, Symptoms, Concerns, Symptom severity, Integrated Palliative care Outcome Scale, Palliative and end of life care, Life-limiting illnesses

## Abstract

**Background:**

Patient-centred measures to capture symptoms and concerns have rarely been reported in severe COVID. We adapted and tested the measurement properties of the proxy version of the Integrated Palliative care Outcome Scale–IPOS-COV for severe COVID using psychometric approach.

**Methods:**

We consulted experts and followed consensus-based standards for the selection of health status measurement instruments and United States Food and Drug Administration guidance for adaptation and analysis. Exploratory Factor Analysis and clinical perspective informed subscales. We tested the internal consistency reliability, calculated item total correlations, examined re-test reliability in stable patients, and also evaluated inter-rater reproducibility. We examined convergent and divergent validity of IPOS-COV with the Australia-modified Karnofsky Performance Scale and evaluated known-groups validity. Ability to detect change was examined.

**Results:**

In the adaptation phase, 6 new items were added, 7 items were removed from the original measure. The recall period was revised to be the last 12–24 h to capture fast deterioration in COVID. General format and response options of the original Integrated Palliative care Outcome Scale were preserved. Data from 572 patients with COVID from across England and Wales seen by palliative care services were included. Four subscales were supported by the 4-factor solution explaining 53.5% of total variance. Breathlessness-Agitation and Gastro-intestinal subscales demonstrated good reliability with high to moderate (a = 0.70 and a = 0.67) internal consistency, and item–total correlations (0.62–0.21). All except the Flu subscale discriminated well between patients with differing disease severity. Inter-rater reliability was fair with ICC of 0.40 (0.3–0.5, 95% CI, *n* = 324). Correlations between the subscales and AKPS as predicted were weak (r = 0.13–0.26) but significant (*p* < 0.01). Breathlessness-Agitation and Drowsiness-Delirium subscales demonstrated good divergent validity. Patients with low oxygen saturation had higher mean Breathlessness-Agitation scores (M = 5.3) than those with normal levels (M = 3.4), t = 6.4 (186), *p* < 0.001. Change in Drowsiness-Delirium subscale correctly classified patients who died.

**Conclusions:**

IPOS-COV is the first patient-centred measure adapted for severe COVID to support timely management. Future studies could further evaluate its responsiveness and clinical utility with clinimetric approaches.

**Supplementary Information:**

The online version contains supplementary material available at 10.1186/s12955-023-02102-4.

## Background

Patients infected with COVID can present with very severe and distressing symptoms including breathlessness and delirium [1], and suffering [2]. Other distressing symptoms such as cough, diarrhoea, fatigue, palpitations and upper airway congestion have also been reported in severe COVID [3]. Individuals reported cognitive deficits, depression and anxiety, loss of smell and loss or distortion of taste, cough, chest pain, fever, fatigue and exhaustion and breathlessness as persistent symptoms [4]. COVID is a life-threatening condition, which have long term debilitating effects [5]. Palliative care as a holistic approach that improves the quality of life of patients and their families facing difficulties associated with life-threatening illness, is relevant to management of symptoms [6] and care of patients severely ill and dying with COVID [7]. Palliative care is concerned with the prevention and relief of suffering by means of early identification, assessment and treatment of physical, psychosocial and spiritual, and is ‘a crucial part of integrated, people-centred health services’ [8].

In severe COVID, deterioration can be sudden, timely recognition of symptoms, management and re-assessment is key. Patient-centred outcome measures (PCOMs) play a key role in informing management and care. COVID and its new variants persist as a threat to public health [9]. A valid and brief PCOM based on life-limiting and advanced illness perspective is relevant and beneficial in COVID.

PCOMs facilitate and add value to care as they provide important evidence to inform decisions about treatment alternatives, decisions, help identify, prioritise, address symptoms, disabilities, aspects of quality of life important for patients. They direct resources to where they are most needed, increase accountability [10] and quality [11] of care. PCOMs in advanced disease may be the only means of capturing the subtle but critical differences interventions make. Capturing needs, concerns, and disease impact directly from the patients, self-reporting, is often considered ideal, but proxy-reports are also valuable [12]. The experience of illness relates to expectations, standards, concerns and is subjective [13]. Self-reporting is challenging and often not possible, for example in patients admitted to intensive care units or referred to palliative care [14–16]. Infection control measures and restrictions on visits mean family or informal carer feedback may not be feasible, hence the importance of proxy-reporting by staff [17].

COVID’s quick progression leading to changes in health status and capacity of the patients to self-report [15] requires considerations when choosing and implementing a PCOM. There are generic [18] as well as condition specific [18, 19] PCOMs in advanced illness. Though broader measures of quality of life and health status are available [20], here we urgently need a symptom measure, adapted to a patient perspective, that has commonality across advanced illness with COVID specific aspects.

The aim of this study is to adapt and validate a relevant and clinically sensible Patient Centred Outcome Measure in patients severely ill and dying of COVID seen by the specialist palliative care services using psychometric methods. Integrated Palliative care Outcome Scale (IPOS) a brief, valid and reliable measure of symptoms and concerns, suitable for self and proxy reporting and widely used across advanced illness, palliative and end of life care [21], was chosen as a relevant measure. Here we describe how we: (1) adapted IPOS to COVID and produced a COVID-specific proxy- reported version called IPOS-COV, and (2) explored IPOS-COV’s components and examined its reliability, validity, and responsiveness among patients with severe COVID.

## Methods

We first adapted the original IPOS to COVID, producing IPOS-COV. Following adaptation, the validation study was conducted on data from the multicentre cohort study of people with severe COVID seen and treated by palliative care services across 25 sites across Wales and England. The study received Health Research Authority (HRA, England) and Health and Care Research Wales (HCRW) approval (REC reference: 20/NW/0259); study co-sponsors: King’s College Hospital NHS Foundation Trust and King’s College London, registered ISRCTN 16561225. The data collection took place in accordance to the Control of Patient Information (COPI) regulation published by the Department of Health and Social Care where healthcare organisations, GPs, local authorities and arm's length bodies were notified that they should share information to support efforts against coronavirus (COVID-19) [22].

### Setting

Specialist palliative care services providing support in hospital, hospice, community, and social settings including care homes across England and Wales that indicated in the first main component of CovPall Study that they were interested in collecting pseudonymised data on a small series of patients with COVID [7].

### Patients

Patients receiving specialist palliative care including those supported via remote consultations, who also either had a test confirmation of COVID, were clinically diagnosed with COVID, or had both test and clinical diagnosis. Patients were aged 18 years or over, with any pre-existing progressive conditions. The services were asked to recruit all eligible patients consecutively as they were admitted to palliative care from February 2020—February 2021 until the target sample of at least 3–5 participants per service was reached according to service size.

### Data collection

First, the experts produced IPOS-COV. IPOS-COV was part of the Case Report Forms (CRF). The finalized CRF was used to collect data. Data was entered retrospectively and prospectively through CRF by the clinical teams responsible for the care of the patient. The site teams also consulted medical reports and notes. A Standardized Operating Procedure (SOP) was prepared detailing the schedule of data collection and data entry. Virtual trainings and meetings were organised to address any questions or site-specific challenges. Sites were sent randomly generated participant ID codes which they assigned to patients. All confidential and sensitive patient data were kept at individual sites.

### Assessments and measures

IPOS-COV, Australia-modified Karnofsky Performance Status (AKPS) and Palliative Phase of Illness (PPoI) were used to capture needs and assess status of patients. In addition, data on demographic as well as key clinical variables were recorded at assessments [15]. Each patient had a baseline assessment on referral, and final assessment at discharge, or in the event of death or at the end of the observation period of study—96 h—if they were still in care.

*Australia-modified Karnofsky Performance Status (AKPS):* AKPS is a clinical rating tool for evaluating a patient’s overall performance status adapted to palliative care and produces a single score between 0 and 100, smaller scores indicate reduced performance status [23].

*Palliative Phase of Illness (PPoI):* Palliative Phase of Illness is a clinical rating tool which describes urgency of care needs where a person is rated as being Stable, Unstable, Deteriorating, Dying or Deceased [24].

*Integrated Palliative/Patient Outcome Scale adapted for COVID (IPOS-COV):* IPOS-COV is 14-item brief PCOM scored on a 5-point Likert scale (0–4), higher scores indicate an overwhelming effect of symptoms and unmet needs.

### Adaptation of IPOS for COVID

Adaptation of IPOS for severe COVID was initiated on 8^th ^of April, 2020, and finalised on 21^st^ of April, 2020. The experts included CovPall Study [25] core team (authors), site teams, study partners (Hospice UK, Marie Curie, Sue Ryder, Palliative Outcome Scale Team, European Association of Palliative Care (EAPC), Together for Short Lives and Scottish Partnership for Palliative Care) and professional network meetings (Hospice UK ECHO Network, KCL Evidence Update, Clinical Academic Group and Researcher’s Exchange Meetings). New items were identified and items from the original measure removed based on available or emerging evidence on symptoms at the beginning of the pandemic by expert consensus. The core structure of IPOS was preserved, in that the emphasis was how a symptom affected the patient, rather than its frequency or severity. The frequency of its reporting and recall period was revised to capture fast deterioration.

### Psychometric testing

Psychometric properties of IPOS-COV were assessed and reported following COSMIN and US FDA guidance for patient reported outcomes [26, 27].

*Identifying IPOS-COV Subscales and Describing their distribution:* Exploratory Factor Analysis (EFA) with principal axis extraction and obligue (direct oblimin) rotation multiple was used to understand symptom clusters and inform subscales [28] in this new illness and patient group. Parallel analysis–examining of the real versus random Eigen values [29]—and clinical judgement were used to identify the most relevant clustering to identify subscales. Once the subscales were identified, acceptability was assessed by examining distribution of item and subscale scores, floor and ceiling effects, data completeness with Missing Value Analysis.

*Reliability:* We evaluated the ability of IPOS-COV to yield consistent, reproduceable estimates of true treatment effects in several ways:

1. we assessed the internal consistency and determined agreement among responses to items. Cronbach’s alpha informed the degree of interrelatedness and agreement among the subscales. The internal consistency coefficient was calculated for each subscale separately as IPOS-COV is multidimensional. Item–total correlations tested the discriminating ability of the items. A correlation coefficient of 0.30, corresponding to a medium effect size, was chosen as the cut-off criterion. An item–total correlation below 0.30 implies that the item cannot discriminate well between patients severely and less severely affected by COVID [30].
2. Test–retest reliability was assessed in patients who were stable, based on PPoI, at baseline and remained stable after 12–24 h in the second assessment, and 24–36 h later in the third assessment. The time interval between the two assessments was 12–24 h, and to the best of our knowledge the evaluation took place under similar care conditions and settings. To demonstrate test–retest reliability, we hypothesised that paired-samples t-test of the subscale scores of patients who remained stable between baseline and follow-up assessments would show no significant difference. The null hypothesis (H_0_) tested here is that the mean difference of subscale scores among baseline and 12–24 h and 24–36 h in follow-up assessments would be 0.
3. We examined inter-rater reproducibility to understand the consistency with which multiple raters assessed patients by using Intraclass correlation coefficient (ICC). The ratings were completed by the clinical teams on each specific site; however, little is known about whether the patients were assessed consistently by the same team members. For this reason, ICC was calculated with One-Way Random model which examines the mean reliability of raters (average measures) and not a single rater [31, 32]. Also, to examine the precision of measurement and determine the effect of measurement error [33], Standard Error of Measurement (SEM) is calculated using the following formula:
  $$SEM\,=\,Standard\, Deviation\sqrt{1-Reliability}$$

SEM between 0.8 and 0.9 is considered evidence of adequate measurement precision [34].

*Construct validity*: We examined the associations between IPOS-COV with AKPS, PPoI and biochemical parameters suggesting severity, and endpoints such as death, according to ‘a priori’ hypotheses. We hypothesized that patients who are more severely affected by COVID (indicated by higher IPOS-COV scores), would have lower functional ability (indicated by lower AKPS scores). We examined divergent validity by hypothesizing those patients with lower oxygen saturation would have higher IPOS-COV scores. Strength and the direction of the association of IPOS-COV with AKPS, IPOS with oxygen levels, were evaluated using Spearman rank-order correlation coefficient.

We formed two groups based on oxygen saturation levels at baseline, categorising patients into ‘low oxygen saturation to include patients with less than 90% oxygen saturation, and ‘high oxygen saturation’ group of 90% and above. We also categorized patients according to those who died and those who were discharged or still in care. We examined discriminative or known-groups validity within these subgroups using independent sample t-tests. We hypothesized that the group of patients with low oxygen saturation, and patients who died to have statistically significant (*p* ≤ 0.05) higher mean IPOS-COV scores compared to those with high oxygen saturation, and those who have been discharged or still in care.

*Responsiveness and Minimally Important Difference:* We examined the ability of IPOS-COV to detect a change in the patient’s status [35] to inform sample size decisions evaluating effectiveness in future trials. We hypothesized that changes in IPOS-COV score would capture improvement or deterioration or would stay the same when there is no change in patient’s status. We first calculated IPOS-COV change scores between (i) baseline (T0) and final (TF), (ii) baseline and time 1 (T1), (iii) time 1 and time 2 (T2) and (iv) time 2 and final assessment. We subtracted the earlier score from that of the later assessment. A positive change score indicates deterioration.

We used clinician rated anchors as well as biochemical markers, and endpoints to define clinical change, as follows:

1. Death as an endpoint was a clinically significant change
2. Patients assessed at baseline to be *unstable*, *deteriorating* or *dying*, who became *stable* in follow-up assessments with PPoI to have *improved* clinically (timepoints for which this data was available is used)
3. Patients who presented with similar levels of C-reactive protein (CRP) at baseline and follow-up assessments were categorised as *unchanged* or *same*. Patients who moved from normal to hyperinflammation were categorised as *deteriorated* and those who have moved from hyperinflammation to normal levels of inflammation, were categorised as clinically *improved* (timepoints for which this data was available is used). (Note: C-reactive protein (CRP) level is an inflammatory biomarker associated with disease development and predictor of severity for COVID [36], where patients with CRP levels below 500 mg/L, had normal levels of inflammation and CRP levels had hyperinflammation and negative clinical course).

Sensitivity and specificity analysis was used to examine if IPOS-COV could correctly identified those who died. The area under curve (AUC) the Receiver Operating Curve (ROC) was examined to test the null hypothesis that AUC would be smaller than or equal to 0.5. ROC curve plot sensitivity (on the y-axis) against 1-specificity (on the x-axis) for all possible cut off points of change scores and relate this to the probability of identifying patients who have died. AUC over 0.70 suggests sufficient responsiveness [27]. AUC of 0.5 suggests IPOS-COV is no better than identifying those who died than a simple guess.

We calculated effect sizes to capture clinically important change [37]. Based on PPoI where patients who showed *improvement* were included in effect size calculations. Effect sizes more than 0.8 are considered large, 0.5 to 0.8 are moderate, between 0.2 to 0.4 are considered as small, and less than 0.2 is considered negligible [38, 39]. The effect sizes were calculated using the formulas below for baseline and time 1 assessment, time 1 and time 2 assessments respectively and were calculated for each of the subscale scores [37]:

$$ES=\frac{{mean \,(baseline-Time \,1)}_{improved}}{{Standard\, Deviation\, of\, baseline\, assessment}_{stable}}$$

$$ES=\frac{{mean \,(Time \,1-Time\, 2)}_{improved}}{{Standard \,Deviation\, of \,Time \,1\, assessment}_{stable}}$$

To capture ability of IPOS-COV to detect change in general, Standardized Responsive Mean (SRM) and the effect sizes for change from baseline to final assessment was calculated using the formula:

$$SRM=\frac{{mean \,(Change\, Score\, from\, baseline\, to\, final)}_{total\, group}}{{Standard\, Deviation\, (Change\, Score\, from\, baseline\, to\, final)}_{total\, group}}$$

Based on the third hypothesis, we calculated Minimally Important Change (MIC) based on median change scores in patients who had *improved* or *deteriorated* between available time intervals.

## Results

### Adaptation of IPOS to COVID

**Fig. 1:**
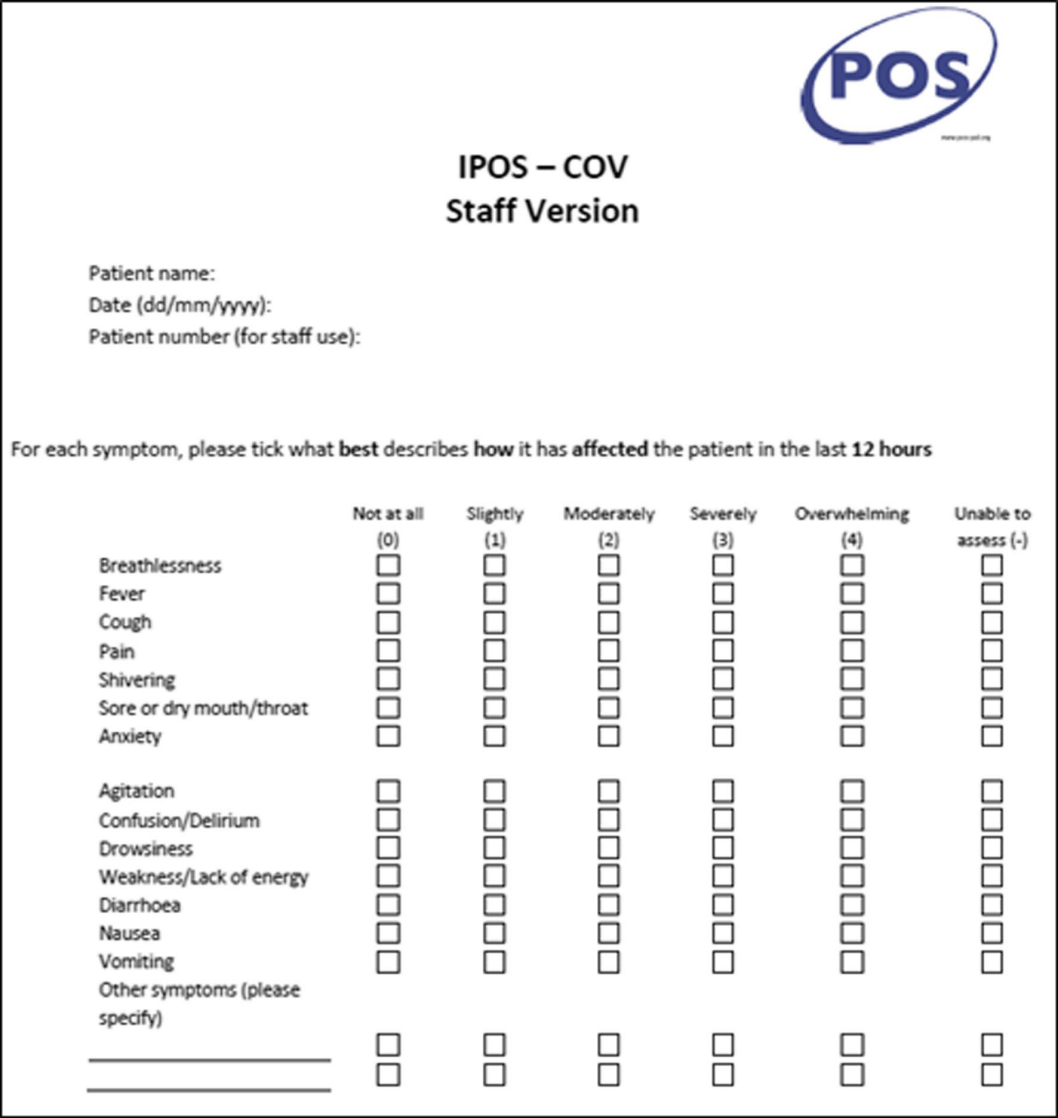
14 item IPOS-COV – brief patient-centred outcome measure for COVID

The CovPall study group included practicing clinicians, developers of the Palliative care Outcome Scale (POS) family of measures [40], as well as members of the POS Development Team. The study team reached consensus on the draft structure and content of IPOS-COV and shared this with wider clinical teams and study partners for feedback. The finalised measure included symptoms such as agitation, confusion/delirium, cough, fever, shivering, diarrhoea as these were reported in symptom profiles of COVID. We also modified ‘sore or dry mouth’ to include ‘sore throat’. ‘Shortness of breath’ was reworded as ‘breathlessness’ to ease proxy reporting. Several items from the original IPOS such as the open-ended question on main concerns, items such as ‘poor appetite’, ‘constipation’, ‘poor mobility’, ‘anxiety of family/friends’, ‘feeling depressed’, ‘feeling at peace’, ‘sharing of feelings with family and friends’, ‘practical problems’, were removed as they were either not relevant or were too subjective and not accessible or observable by proxies. The recall period was changed from the original three days recommended in acute settings to ‘the last 12 h.’ We added ‘unable to assess’ as a response option (Fig. 1).

### Psychometric testing

Table 1 describes the 572 study participants. They had poor performance status with median AKPS score of 20 (bedfast). Few of the patients who were admitted were stable, and most died at the end of the follow-up period. Further information about the patients is detailed elsewhere [15].

**Table 1:** Demographic and clinical characteristics of the participants (*n* = 572)

Characteristics / variable	Total sample (*n* = 572^a^)
Age, Mean (Median, Range)	77.2 (80, 32 to 102)
Sex, Female *n* (%)	264 (46.2)
*Ethnicity, *n* (%)*	
White (British and Other)	436 (79.9)
Other^b^	110 (20.1)
*BMI, *n* (%)*	
Underweight (< 18.5)	96 (20.3)
Healthy (24.9–18.5)	201 (42.4)
Overweight (29.9–25)	110 (23.2)
Obese (> 30)	67 (14.1)
*Status at Baseline, *n* (%)*	
New to Palliative Care services	75 (13.1)
Already supported by palliative care services	496(86.9)
Number of IPOS-COV symptoms recorded at Baseline Mean (Median, Range)	4.9 (5, 0 to 12)
Baseline AKPS Score, Mean (Median, Range)	24.3 (20, 10 to 90)
*Assessments:*	
Baseline Oxygen Saturation (%) Mean (Median, Range)	90.4 (93,48 to 100)
Baseline C-reactive Protein, *n*, Mean (Median, Range) mg/L	436, 141.4 (120.5, 2 to 449)
Baseline D-dimer test value^c^, *n*, Mean (Median, Range) µg/L	103, 3679.3 (1630, 36.1 to 67,010)
Baseline Lymphocyte count^d^, *n*, Mean (Median, Range(10^9^L)	1 (0.8, 0.1 to 17.5)
Baseline Lactate Dehydrogenase^e^, *n*, Mean (Median, Range) U/L	57, 1483 (539, 111 to 43,893)
*Phase of Illness at Baseline Assessment n (%)*	
Stable	17 (3)
Unstable	171 (30.3)
Deteriorating	151 (26.7)
Dying	226 (40)
*Outcome at the end of the study observation and follow-up period, n (%)*	
Died	417 (73)
Discharged or Still in Care	154 (27)
*Patients admitted at waves of COVID*^*f*^*, n (%)*	
Wave 1 (February 2020—August 2020)	316 (61.4)
Wave 2 (September 2020—February 2021)	199 (38.6)

^a^1 case is missing most of the demographic and clinical information, most present findings from *n* = 571

^b^includes Asian/Asian British, Black/African/Caribbean/Black British, Arab, Mixed/Multiple ethnic group

^c^5 cases with D-dimer values that did not seem possible are not presented

^d^2 cases with Lymphocyte values that did not seem possible are not presented

^e^20 cases with LDH values that did not seem possible are not presented

^f^To define the UK pandemic waves we used approaches taken by the King’s Fund and the Office for National Statistics (references)

*IPOS-COV Subscales and Distribution of Scores:* Parallel analysis initially suggested a 2-factor solution (Additional file 1: Figure S1). However, following examination of 2 to 4 factor solutions, item communalities, and factor loadings, a 4-factor solution explaining 53.5% of the total variance was clinically most relevant while fitting the data (Table 2). ‘Diarrhoea’ was removed as it failed to load onto any of the factors.

**Table 2:** IPOS-COV subscales items, factor loadings and percentage variance explained by each factor/subscale

Items	Breathlessness and Agitation (Breath-Ag)	Gastro-intestinal Issues (GI)	Drowsiness and Delirium (Drow-Deli)	Flu-like symptoms (Flu)
(Variance Explained %)	18.3	13.7	10.9	10.6
Agitation	**0.81**			
Anxiety	**0.72**			
Breathlessness	**0.53**			
Nausea		**0.90**		
Vomiting		**0.61**		
Drowsiness			**0.85**	
Weakness or lack of energy			**0.47**	
Confusion or Delirium			**0.33**	
Sore or dry mouth or throat				**0.47**
Fever				**0.41**
Cough				**0.37**
Shivering				**0.35**
Pain				**0.30**

Blanks represent factor loadings < 0.30/ − 0.30. Numbers in bold represent factor loadings.

Missing values were highest for the IPOS-COV anxiety item (Additional file 1: Table S1). Item floor effects (< 15%) are acceptable, all have ceiling effects. The subscales have acceptable ceiling effects; GI and Flu have floor effects (Additional file 1: Table S1).

*Reliability* Breathlessness-Agitation (Breath-Ag) subscale (α = 0.70) and the Gastro-Intestinal (GI) Subscale (α = 0.67) show high to moderate internal consistency reliability. In contrast, the Drowsiness-Delirium (Drow-Deli) subscale (α = 0.55), and Flu subscale (α = 0.42) has low internal consistency. Most item–total correlations are 0.30 and higher, demonstrating that they discriminate well between persons at different levels of severity (Additional file 1: Table S2). Test–retest reliability is inconclusive as too few patients remained stable in follow-up assessments for analysis (Additional file 1: Table S3). Inter-rater reliability is fair with ICC of 0.40 (0.302—0.494 95%CI, *n* = 324) for average measures. Measurement accuracy is low (SEM = 4.1).

*Construct Validity* Correlations between IPOS-COV subscales and AKPS were weak (*r* = 0.13–0.26) but significant (< 0.01) (Table 3). Participants with better performance status were affected less by symptoms such as agitation and drowsiness as hypothesized; patients with better functional status were affected more by symptoms such as fever and nausea.

**Table 3:** Convergent and Divergent Validity-Association of IPOS-COV subscale scores with AKPS (Spearman rank-order correlation coefficient)

IPOS-COV Subscales	AKPS		
	*P* Sig(2-tailed)	Spearman's Rho	*n*
Breath-Ag	< 0.01	− 0.13	431
GI	< 0.001	0.23	485
Drow-Deli	< 0.001	− 0.26	446
Flu	< 0.001	0.25	421

The Breath-Ag subscale had discriminative validity between those participants with low oxygen saturation at baseline, who had significantly higher mean subscale scores (M = 5.3), compared to those with normal oxygen saturation (M = 3.4), t = 6.4 (186), *p* < 0.001 (Additional file 1: Table S4). Participants with normal oxygen saturation experienced significantly higher GI subscale scores compared to those with lower oxygen saturation. With the Flu and Drow-Deli subscales the pattern was not clear.

*Ability to detect change and Minimally Important Difference* Participants who died had higher mean and median Breath-Ag and Drow-Deli subscales (Table 4, Additional file 1: Table S5, Fig. S2 a-d), providing evidence these two subscales are responsive to the changes in status of patients.

**Table 4:** Baseline and final assessments according to outcome at the end of the observation period of the study (*n* = 572)

	Outcome at the end of the observation period of the study	
	Discharged or still in care (*n* = 154)	Died (*n* = 417)
Sex, Female (%)	51.9	44.1
Age Mean (Median, Range)	75.1 (76.5,32 to 100)	78 (80, 34 o 102)
*Baseline (T0) Mean* ± *SD (Median, Range)*		
Breathlessness and Agitation	2.5 ± 2.2 (2, 0 to 9)	4.4 ± 2.9 (4, 0 to 12)
Gastro-intestinal Issues	0.2 ± 0.7 (0, 0 to 4)	0.2 ± 0.8 (0, o to 12)
Drowsiness and Delirium	3.8 ± 2.4 (3, 0 to 12)	4.7 ± 2.7 (4, 0 to 12)
Flu-like symptoms	2.6 ± 2.1 (2, 0 to 9)	2.4 ± 2.4 (2, 0 to 12)
*Final (TF) (Mean, Median, Range)*		
Breathlessness and Agitation	1.6 ± 2.2 (1, 0 to 12)	2.6 ± 2.5 (2. 0 to 12)
Gastro-intestinal Issues	0.2 ± 0.7 (0, 0 to 4)	0 ± 0.1 (0, 0 to 2)
Drowsiness and Delirium	3.4 ± 2.6 (3, 0 to 10)	5.2 ± 3.2 (6, 0 to 12)
Flu-like symptoms	1.3 ± 1.8 (0, 0 to 9)	1 ± 1.7 (0, 0 to 10)

The observations may be different groups, the comparisons are not paired, the patients in baseline group may not all have Time 1 or Time 2 data, but most have Final assessment data

**Fig. 2:**
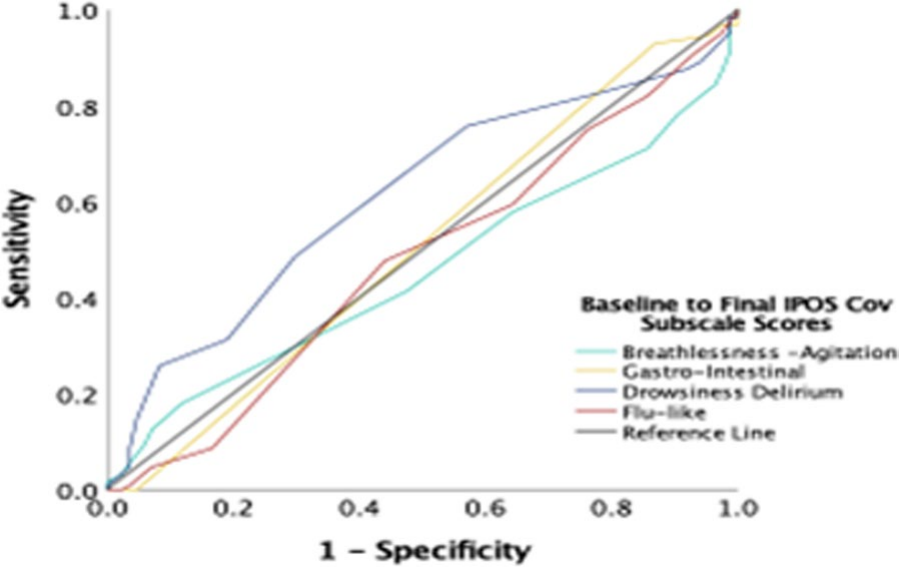
Receiver Operating Characteristic (ROC) curves for IPOS-COV subscale baseline-final change scores (*n* = 212)

Sensitivity and specificity analysis also shows that change in Drow-Deli subscale scores correctly classify patients who died (Fig. 2, Additional file 1: Fig. S3 a-d, Table S6).

SRM suggest that Breath-Ag and Flu subscales detect change, having moderate effect sizes for change from baseline to final assessment (Table 5).

**Table 5:** Standardized Response Means (SRM) for the total sample for change scores between baseline and final assessments

Subscale	SRM	Mean (Change Scores)	SD (Change Score)	*n*
Breath-Ag	− 0.5	− 1.4	2.9	342
GI	− 0.2	− 0.2	0.7	384
Drow-Deli	0.0	0.1	3.2	339
Flu	− 0.6	− 1.3	2.1	325

Effect sizes were small to moderate in the Breath-Ag and GI subscales, and negligible in the Drow-Deli and Flu subscales, in the small number of patients who had improved between baseline and first follow up assessments (Additional file 1: Table S7). Effect sizes were moderate to high for all subscales except for the GI and Flu subscales between first and second follow-up assessments (Additional file 1: Table S7). Findings on MIC are inconclusive (Additional file 1: Table S8).

## Discussion

IPOS-COV is a patient-centred outcome tool that can be used for timely monitoring and recognition, management and re-assessment of key symptoms of patients severely ill with COVID. It can be used to quantify severity of distressing symptoms. IPOS-COV can also be used to identify patients presenting with a complex cluster of symptoms to direct fast and effective care to them.

IPOS-COV is a 14-item brief multi-dimensional tool, adapted for proxy-reporting. IPOS-COV has four clinically relevant subscales: (1) the Breathlessness-Agitation (Agitation, Anxiety and Breathlessness), (2) Gastro-intestinal (Nausea and Vomiting, (3) Drowsiness-Delirium (Drowsiness, Weakness or lack of energy, Confusion or Delirium) and (4) Flu (Sore or dry mouth or throat, Fever, Cough, Shivering, Pain). A total score can be calculated by summing all the item scores. Subscale scores can be obtained by summing of items within a subscale, for example the Breathlessness and Agitation subscale score is calculated by summing item scores of Agitation, Anxiety and Breathlessness. Individual item scores can also be used to monitor certain symptoms or identifying predictors of clinical outcomes [15].

IPOS-COV includes and focuses on the symptoms reported to be most distressing and prevalent in COVID, such as breathlessness and agitation [41], in patients too unwell to self-report. Some of the symptoms included in IPOS-COV such as breathlessness and agitation, have been recognised as needing the most urgent attention [42].

It is an acceptable tool and valid in severe COVID. Its items are clear, and concepts are accessible. Its implementation is feasible, and acceptable in clinical settings. The completion rates, and the feedback from the study sites suggests that IPOS-COV is easy to use in research and practice, however further studies are needed to formally evaluate IPOS-COV’s clinical utility.

When adapting IPOS to COVID, 6 new items were added, 7 were removed and the recall period revised to ‘last 12–24 h’ to capture fast deterioration. The general IPOS structure and format were preserved. The adaptation of IPOS, to severe COVID did not include input from patients, and only included expert feedback from the core team, site teams and partner organisations. Available or emerging evidence on symptoms at the beginning of the pandemic were reviewed. The consensus approach used elements of Consensus Development Conference, where face-to-face discussions were held [43]. Future studies could include patient feedback.

IPOS-COV has structural validity with mostly moderate to high factor loadings. Acceptability of IPOS-COV is high. Anxiety had high missingness as this is symptom is less observable or accessible to proxies. The Breathlessness-Agitation and Drowsiness-Delirium subscales have acceptable floor effects. High ceiling effects have been only observed with the Gastro-Intestinal subscale. Most of the patients in the cohort, have high scores on this subscale. Gastrointestinal symptoms such as diarrhoea, nausea and vomiting are frequently reported in COVID [44]. For this reason, the Gastro-Intestinal subscale may not be useful in informing resource allocation decisions or prioritisation in this cohort of patients. Scale calibration, or data transformation are recommended with continuous scales, however careful consideration is needed with ordinal scales such as with IPOS-COV [45]. Choosing rank-based non-parametric statistical analysis methods may reduce the impact of the ceiling effects on findings [45].

The Breathlessness-Agitation and Gastro-Intestinal subscales show moderate to high internal consistency reliability. The high item–total correlations shows that IPOS-COV subscales discriminate well between persons less and those more severely affected by COVID. Presentation of multiple severe symptoms in a patient may complicate clinical monitoring and management decisions. Nationally implemented tools are available to support clinical monitoring [46], but there may be limitations to their applicability in severe COVID [47]. IPOS-COV and its two subscales could support clinical decision making, where patients who are assessed to have higher scores on Breathlessness-Agitation and Gastro-Intestinal subscales could be identified as more severe, needing a fast and efficient clinical response.

Patients with higher Breathlessness-Agitation subscales scores, are also reported to have poorer AKPS scores, thus poorer performance status. This observation suggests that IPOS-COV has convergent validity, and IPOS-COV Breathlessness-Agitation subscale addresses a similar content and construct as AKPS. The Breathlessness-Agitation and Drowsiness-Delirium subscales are responsive to clinically important change, and the Drowsiness-Delirium subscale also shows sensitivity and specificity to clinical changes in the patient. These findings suggest that changes in the Drowsiness-Delirium subscale may be used to predict clinical outcomes, where a positive change in Drowsiness-Delirium subscale score could be an indicator of deterioration, and poor outcomes.

In this study, we demonstrate that implementation of IPOS-COV as a brief and multidimensional measure is feasible. A comprehensive health-related quality of life measure for patient-reporting in COVID has recently been developed [48]. Survey fatigue in severely ill patients and unsustainability of high provider and staff engagement in intensive or critical care settings may affect feasibility of long measures [49], and may limit clinical utility. When people are severely ill, a proxy-reported measure based on the main symptoms and concerns which patients report, is important.

The study has certain limitations. The sample included patients who were seriously ill and dying with COVID, and patient-reporting was not feasible. For this reason, clinical anchors and judgement were used to identify patients who had shown improvement or remained stable over time. Limited numbers of patients had shown improvement or remained stable; therefore, it was not possible to evaluate aspects such as reproducibility of IPOS-COV overtime and quantify minimum clinically important differences. Certain items that were more difficult to observe such as agitation generated higher missingness than more observable items such as breathlessness. Identification of equivalent items was not possible as IPOS-COV is brief and reducing respondent burden was prioritized. Also, approaches that would have reduced missingness such as use of stringent proxy inclusion criteria was not an option in COVID [50]. The analysis therefore excluded cases with missing data, and this may have introduced bias. Multiple imputation at the item level could be explored with IPOS-COV in future studies, specifically in clinical trials [51].

The study undertook the evaluation of psychometric properties of IPOS-COV, and presents the findings based on psychometric framework. The study presents some evidence of IPOS-COV’s clinical utility, and specifically sensitivity. Due to the unprecedented strain the health and social care services were under during the COVID pandemic, IPOS-COV’s ease of use and format were considered thoroughly. Further evidence of IPOS-COV’s incremental validity is reported elsewhere [15]. Future studies and further analysis using clinimetric approach is needed to provide further evidence and insights into its clinical utility in health and social care settings [52].

One of the strengths of the study is that it includes data from a large cohort of patients with complex clusters of symptoms, with and without co-morbidities, cancer, and non-cancer populations, patients new to and those already supported by palliative care [15]. This study allows us to recognise COVID as an advanced and life-limiting illness, and to understand that symptoms such as fever and shivering frequently reported in COVID [1], may not be relevant in patients with severe COVID.

## Conclusion

IPOS-COV is a robust and brief patient-centred proxy-rated tool specifically adapted and validated in severe COVID using psychometric approach. IPOS-COV may support case management and monitoring of patients severely ill with COVID, furthering our understanding of PCOMs in a new illness, as well as proxy-reporting when patients are too unwell to self-report.

Reproducibility, responsiveness, and Minimally Important Clinical change of IPOS-COV need to be further assessed. Relevance and validity of IPOS-COV in patients infected with new emerging variants could also be explored. Studies to understand and evaluate IPOS-COV’s clinical utility in health and social care settings is warranted.

## Supplementary Information

**Additional file 1. ** Supplementary Tables and Figures.

## Data Availability

Due to ethical concerns, supporting data cannot be made openly available. Please contact the senior author and the Chief Investigator of the study Professor Irene J Higginson (irene.higginson@kcl.ac.uk) for further information about the data and conditions for access.

## Abbreviations

AKPS: Australia-modified Karnofsky Performance Status
COSMIN: Checklist for evaluating the methodological quality of studies on measurement properties
COVID: Coronavirus disease
CRF: Case report forms
EFA: Exploratory factor analysis
HCRW: Health and care research wales
HRA: Health research authority
ICC: Intraclass correlation coefficient
IPOS: Integrated palliative care/Patient outcome scale
IPOS-COV: Integrated palliative care/Patient Outcome Scale adapted for COVID
PCOMs: Patient-centred outcome measures
PPoI: Palliative phase of illness
ROC: Receiver operating curve
SOP: Standardized operating procedure
US FDA: United States food and drug administration

## Declarations

### Availability of supporting data

Due to ethical concerns, supporting data cannot be made openly available. Please contact the senior author and the Chief Investigator of the study Professor Irene J Higginson (irene.higginson@kcl.ac.uk) for further information about the data and conditions for access.
