# Supplementary material for: Adaptation and multicentre validation of a patient-centred outcome scale for people severely ill with COVID (IPOS-COV)
Source: Health Qual Life Outcomes. 2023 Mar 24;21:29. doi: 10.1186/s12955-023-02102-4 (PMC10036974; doi:10.1186/s12955-023-02102-4)
Supplement: Supplementary file 1 — Additional file 1. Supplementary Tables and Figures. [file 12955_2023_2102_MOESM1_ESM.docx]

**Supplementary Information**

**Additional File 1: Supplementary Materials**

[Table S 1 Distribution of item and subscale scores, data completeness, floor, and ceiling effects in baseline assessment 3](#_Toc114568372)

[Table S 2 Reliability: Item-to-Total Correlations 3](#_Toc114568373)

[Table S 3 Test-retest reliability - Comparison of means of IPOS-COV subscales with paired t-test (n=10) of a subgroup of patients who remained ‘stable’ T1 and T2 after T0 4](#_Toc114568374)

[Table S 4 Independent Samples t-test of baseline IPOS-COV subscales scores comparing patients with low and normal baseline oxygen saturations 4](#_Toc114568375)

[Table S 5 Baseline and follow-up assessments according to outcome at the end of the observation period of the study (n=572) 5](#_Toc114568376)

[Table S 6 Area Under the Curve, 95% Confidence Intervals for IPOS-COV subscale change scores for different intervals (significant differences, p<0.05 are in bold) 7](#_Toc114568377)

[Table S 7 Effect sizes in patients who have improved according to PPoI 9](#_Toc114568378)

[Table S 8 Minimum Importance Change (MIC) in patients who improved or deteriorated according to CRP levels 10](#_Toc114568379)

**List of Figures**

[Figure S 1 Plot of real versus random eigen values generated by Parallel Analysis supporting a 2-factor solution, Exploratory Factor Analysis with principal axis extraction and obligue (direct oblimin) rotation ((n=572) 2](#_Toc114568453)

[Figure S 2 Clustered Boxplot of IPOS-COV subscales according to outcome at the end of the study and whether they are new or known to palliative care 6](#_Toc114568454)

[Figure S 3 Receiver Operating Characteristic (ROC) curves for IPOS-COV subscale change scores (baseline and follow-up time-points) 8](#_Toc114568455)

Figure S 1 Plot of real versus random eigen values generated by Parallel Analysis supporting a 2-factor solution, Exploratory Factor Analysis with principal axis extraction and obligue (direct oblimin) rotation ((n=572)


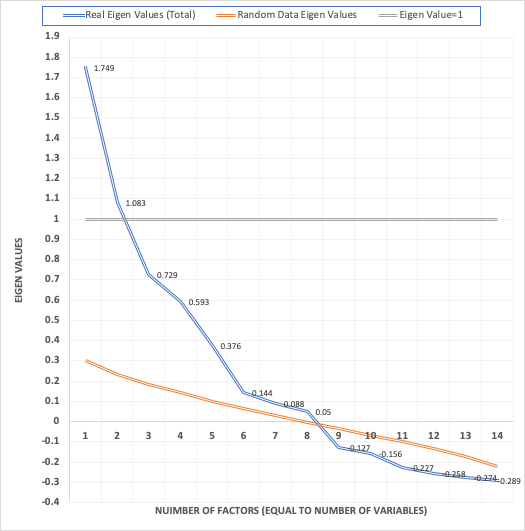


Table S 1 Distribution of item and subscale scores, data completeness, floor, and ceiling effects in baseline assessment

| **Baseline** | | | | | | |
| --- | --- | --- | --- | --- | --- | --- |
| **Item/Subscale** | *n* | *Mean* | *SD* | *Missing (%)* | *Floor Effect (%)* | *Ceiling Effect (%)* |
| Breathlessness | 542 | 1.8 | 1.2 | 5.2 | 19.4 | 7.4 |
| Weakness / Lack of energy | 506 | 2.4 | 1.2 | 11.5 | 12.8 | 13.4 |
| Drowsiness | 523 | 1.4 | 1.3 | 8.6 | 36.5 | 7.6 |
| Anxiety | 471 | 1.1 | 1.2 | 17.7 | 45.9 | 2.1 |
| Agitation | 506 | 1 | 1.2 | 11.5 | 52.8 | 2.2 |
| Confusion/Delirium | 486 | 0.9 | 1.1 | 15 | 56.6 | 1 |
| Cough | 515 | 0.6 | 0.9 | 10 | 62.9 | 0.2 |
| Pain | 527 | 0.8 | 1 | 7.9 | 56.9 | 0.9 |
| Sore or dry mouth/throat | 490 | 0.7 | 1 | 14.3 | 59.8 | 1.2 |
| Fever | 529 | 0.5 | 0.9 | 7.5 | 74.3 | 0.4 |
| Shivering | 498 | 0.1 | 0.3 | 14.3 | 96.4 | 0 |
| Diarrhoea | 518 | 0.1 | 0.4 | 9.4 | 94.6 | 0 |
| Nausea | 492 | 0.2 | 0.5 | 14 | 90.7 | 0 |
| Vomiting | 530 | 0.1 | 0.3 | 7.3 | 96.4 | 0 |
| Breath-Ag | 432 | 3.9 | 2.8 | 24.5 | 14.1 | 0 |
| GI | 486 | 0.2 | 0.7 | 15 | 90.7 | 0 |
| Drow-Deli | 447 | 4.4 | 2.6 | 21.9 | 7.4 | 0.4 |
| Flu | 422 | 2.4 | 2.3 | 26.2 | 26.5 | 0 |

Table S 2 Reliability: Item-to-Total Correlations

| **Subscales** | **Items** | **Corrected Item-Total Correlation** |
| --- | --- | --- |
| **Breath-Ag** | Breathlessness | 0.43 |
|  | Anxiety | 0.59 |
|  | Agitation | 0.53 |
| **GI** | Nausea | 0.62 |
|  | Vomiting | 0.62 |
| **Drow-Deli** | Weakness or lack of energy | 0.37 |
|  | Drowsiness | 0.52 |
|  | Confusion or Delirium | 0.23 |
| **Flu** | Sore or dry mouth or throat | 0.21 |
|  | Fever | 0.23 |
|  | Cough | 0.25 |
|  | Shivering | 0.26 |
|  | Pain | 0.24 |

Table S 3 Test-retest reliability - Comparison of means of IPOS-COV subscales with paired t-test (n=10) of a subgroup of patients who remained ‘stable’ T1 and T2 after T0

| IPOS-COV Subscales | Mean Difference | Std. Deviation | Std. Error Mean | 95% Confidence Interval of the Difference | | t | df | Sig. (2-tailed) |
| --- | --- | --- | --- | --- | --- | --- | --- | --- |
|  |  |  |  | Lower | Upper |  |  |  |
| **Breathlessness and Agitation** | -0.10 | 1.10 | 0.35 | -0.89 | 0.69 | -0.29 | 9 | 0.78 |
| **Gastro-intestinal Issues** | 0.63 | 1.50 | 0.38 | -0.17 | 1.42 | 1.67 | 15 | 0.12 |
| **Drowsiness and Delirium** | -0.38 | 1.50 | 0.42 | -1.29 | 0.52 | -0.92 | 12 | 0.37 |
| **Flu-like symptoms** | 0.25 | 1.36 | 0.39 | -0.61 | 1.11 | 0.64 | 11 | 0.54 |

Table S 4 Independent Samples t-test of baseline IPOS-COV subscales scores comparing patients with low and normal baseline oxygen saturations

|  | **Mean Oxygen Saturation %** | |  |  | |  |  |  |  |
| --- | --- | --- | --- | --- | --- | --- | --- | --- | --- |
| **IPOS-COVID** | **<90% (n)** | **≥90%(n)** | **Mean Difference** | **95%CI of the Difference** | | **t** | **df** | **p-value (2-tailed)** | **Cohen's d (point estimate for effect size)** |
|  |  |  |  | **Lower** | **Upper** |  |  |  |  |
| Breath-Ag | 5.3(107) | 3.4(325) | 1.9 | 1.3 | 2.5 | 6.4 | 186 | **<0.001** | 0.7 |
| GI | 0.1(119) | 0.2(367) | -1.3 | -2.6 | 0 | -2.1 | 298 | **<0.05** | -0.2 |
| Drow-Del | 4.1(111) | 4.5(336) | -1.6 | -1 | 0.1 | -1.6 | 198 | 0.11 | -0.2 |
| Flu | 2.3(96) | 2.5(326) | -0.2 | -0.8 | 0.3 | -0.9 | 151 | 0.37 | -0.1 |
| Equal variance not assumed and significant differences in bold | | | | | | | | | |

Table S 5 Baseline and follow-up assessments according to outcome at the end of the observation period of the study (n=572)

|  | **Outcome at the End of the Observation Period of the Study** | |
| --- | --- | --- |
|  | **Discharged or Still in Care (n=154)** | **Died (n=417)** |
| **Sex, Female (%)** | 51.9 | 44.1 |
| **Age Mean (Median, Range)** | 75.1 (76.5,32 to 100) | 78 (80, 34 o 102) |
| **Baseline (T0) Mean±SD (Median, Range)** |  |  |
| Breathlessness and Agitation | 2.5±2.2 (2, 0 to 9) | 4.4±2.9 (4, 0 to 12) |
| Gastro-intestinal Issues | 0.2±0.7 (0, 0 to 4) | 0.2±0.8 (0, o to 12) |
| Drowsiness and Delirium | 3.8±2.4 (3, 0 to 12) | 4.7±2.7 (4, 0 to 12) |
| Flu-like symptoms | 2.6±2.1 (2, 0 to 9) | 2.4±2.4 (2, 0 to 12) |
| **Time 1 (T1) (Mean, Median, Range)** |  |  |
| Breathlessness and Agitation | 1.9±1.9 (1.5, 0 to 7) | 3.4±2.7 (3, 0 to 12) |
| Gastro-intestinal Issues | 0.2±0.9 (0, 0 to 6) | 0.1±0.6 (0, 0 to 5) |
| Drowsiness and Delirium | 3.5±2.3 (3, 0 to 9) | 5.2±3 (5, 0 to 12) |
| Flu-like symptoms | 2.2±2.2 (2, 0 to 8) | 1.8±1.9 (1, 0 to 9) |
| **Time 2 (T2) (Mean, Median, Range)** |  |  |
| Breathlessness and Agitation | 1.8±2 (1, 0 to 9) | 2.8±2.5 (2, 0 to 10) |
| Gastro-intestinal Issues | 0.3±0.9 (0, 0 to 6) | 0.1±0.4 (0, 0 to 4) |
| Drowsiness and Delirium | 3.6±2.4 (3, 0 to 10) | 5.2±2.9 (5, 0 to 11) |
| Flu-like symptoms | 1.8±2 (1, 0 to 8) | 1.5±1.9 (1, 0 to 9) |
| **Final (TF) (Mean, Median, Range)** |  |  |
| Breathlessness and Agitation | 1.6±2.2 (1, 0 to 12) | 2.6±2.5 (2. 0 to 12) |
| Gastro-intestinal Issues | 0.2±0.7 (0, 0 to 4) | 0±0.1 (0, 0 to 2) |
| Drowsiness and Delirium | 3.4±2.6 (3, 0 to 10) | 5.2±3.2 (6, 0 to 12) |
| Flu-like symptoms | 1.3±1.8 (0, 0 to 9) | 1±1.7 (0, 0 to 10) |
| Note: The observations may be different groups, the comparisons are not paired, the patients in baseline group may not all have Time 1 or Time 2 data, but most have Final assessment data | | |

Figure S 2 Clustered Boxplot of IPOS-COV subscales according to outcome at the end of the study and whether they are new or known to palliative care

1. Baseline Breath-Ag Subscale Scores


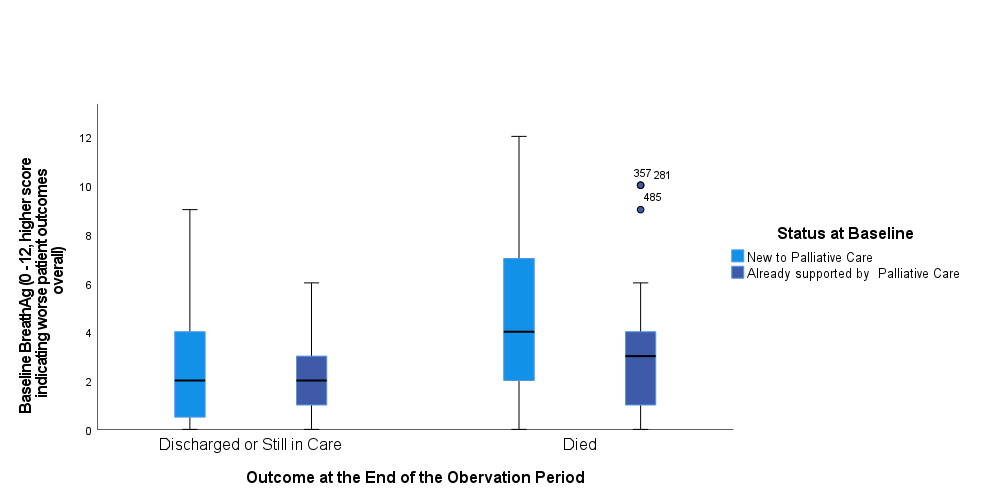


1. Final Breath-Ag Subscale Scores
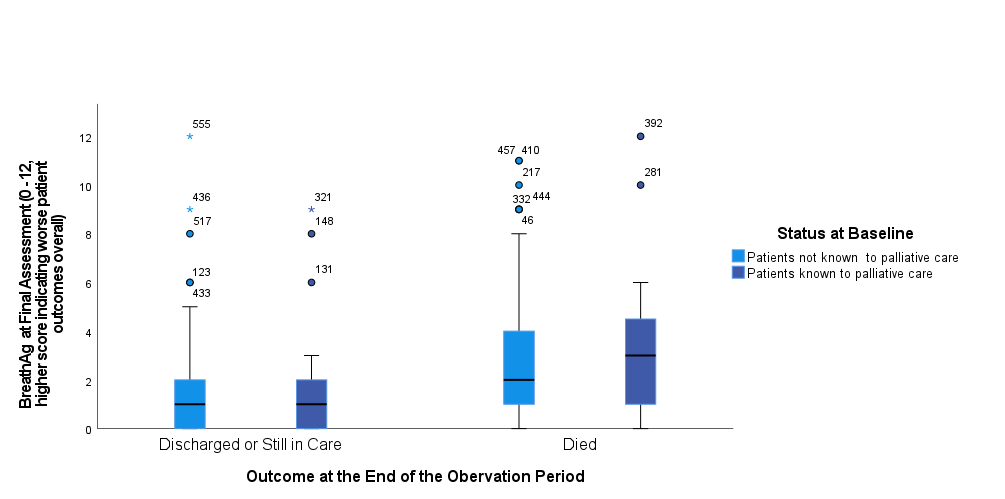


(c) Baseline Drow-Deli Subscale Scores


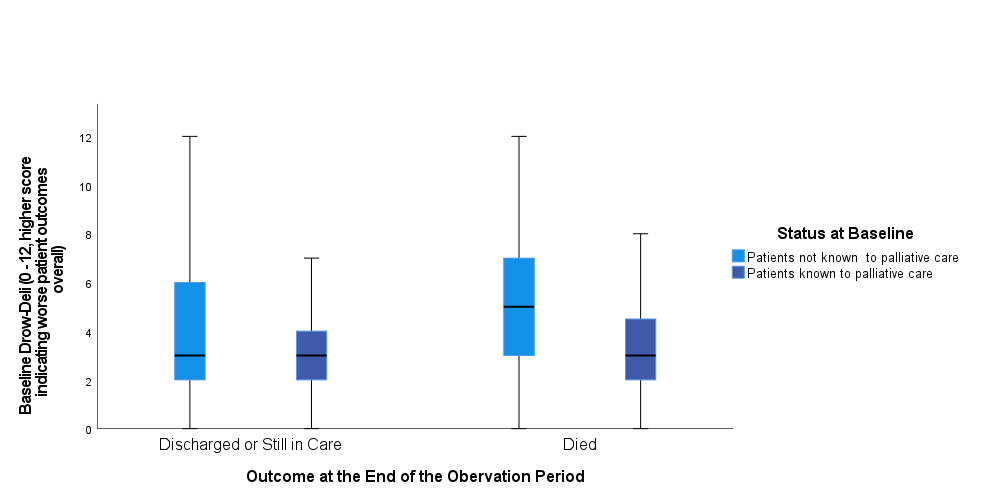


(d) Final Drow-Deli Subscale Scores


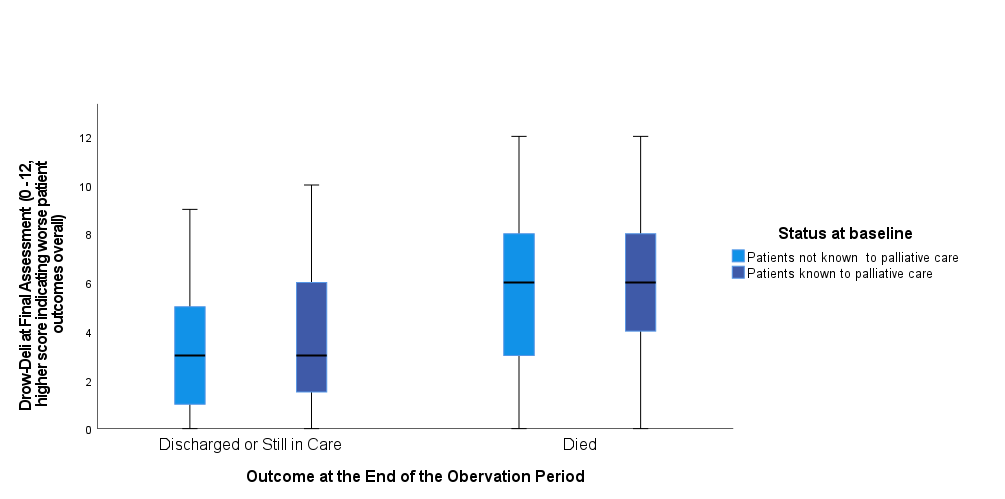


Table S 6 Area Under the Curve, 95% Confidence Intervals for IPOS-COV subscale change scores for different intervals (significant differences, p<0.05 are in bold)

| **Time interval** | **Subscales** | **AUC** | **SE** | **Asymptotic Sig.**  **(p-value)** | **Asymptotic 95% Confidence Interval** | |
| --- | --- | --- | --- | --- | --- | --- |
|  |  |  |  |  | ***Lower Bound*** | ***Upper Bound*** |
| Baseline - Final | Breath-Ag | 0.46 | 0.04 | 0.27 | 0.38 | 0.53 |
|  | GI | 0.51 | 0.04 | 0.89 | 0.43 | 0.59 |
|  | **Drow-Deli** | **0.61** | **0.04** | **0.01** | **0.54** | **0.69** |
|  | Flu | 0.48 | 0.04 | 0.56 | 0.40 | 0.56 |
| Time 1 - Baseline | Breath-Ag | 0.46 | 0.04 | 0.27 | 0.38 | 0.53 |
|  | GI | 0.51 | 0.04 | 0.89 | 0.43 | 0.59 |
|  | **Drow-Deli** | **0.61** | **0.04** | **0.01** | **0.54** | **0.69** |
|  | Flu | 0.48 | 0.04 | 0.56 | 0.40 | 0.56 |
| Time 1 - Time 2 | Breath-Ag | 0.46 | 0.05 | 0.39 | 0.37 | 0.55 |
|  | GI | 0.50 | 0.05 | 0.96 | 0.41 | 0.59 |
|  | Drow-Deli | 0.51 | 0.05 | 0.85 | 0.42 | 0.60 |
|  | Flu | 0.48 | 0.05 | 0.64 | 0.39 | 0.57 |
| Time 2 - Final | Breath-Ag | 0.44 | 0.05 | 0.21 | 0.34 | 0.54 |
|  | GI | 0.51 | 0.05 | 0.92 | 0.41 | 0.60 |
|  | Drow-Deli | 0.58 | 0.05 | 0.10 | 0.48 | 0.67 |
|  | Flu | 0.48 | 0.05 | 0.60 | 0.38 | 0.57 |

Figure S 3 Receiver Operating Characteristic (ROC) curves for IPOS-COV subscale change scores (baseline and follow-up time-points)

1. **Baseline to Final (n=212)**

**
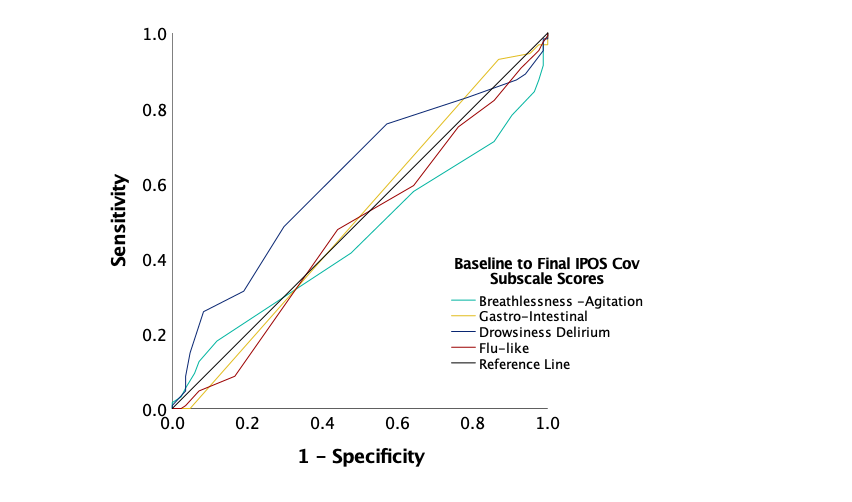
**

1. **Baseline to Time 1 (n=196)**

**
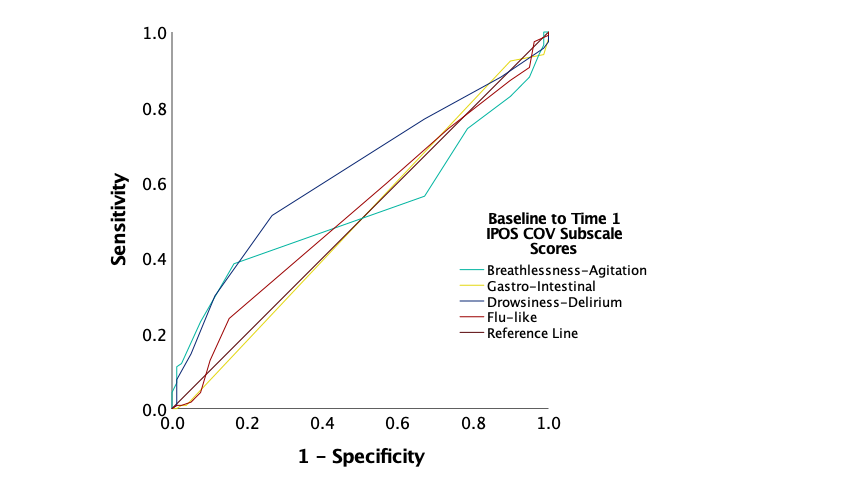
**

1. **Time 1 to time 2 (n=155)**

**
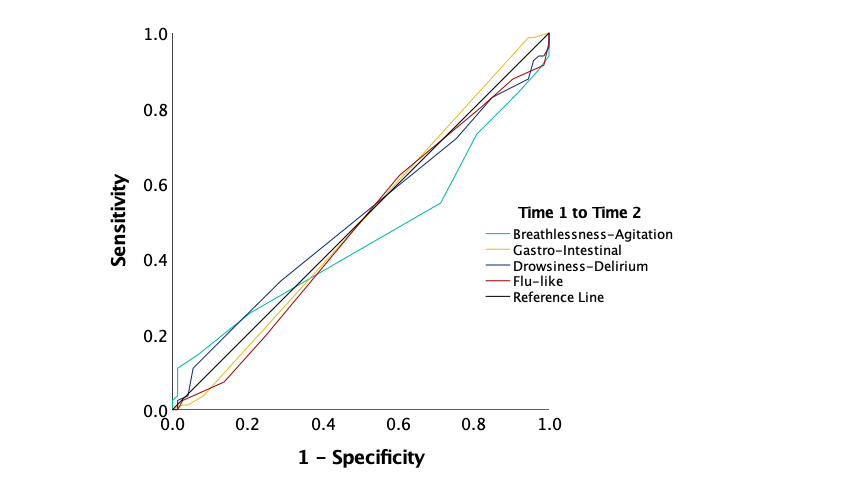
**

1. **Time 2 and Final assessment (n=148)**

**
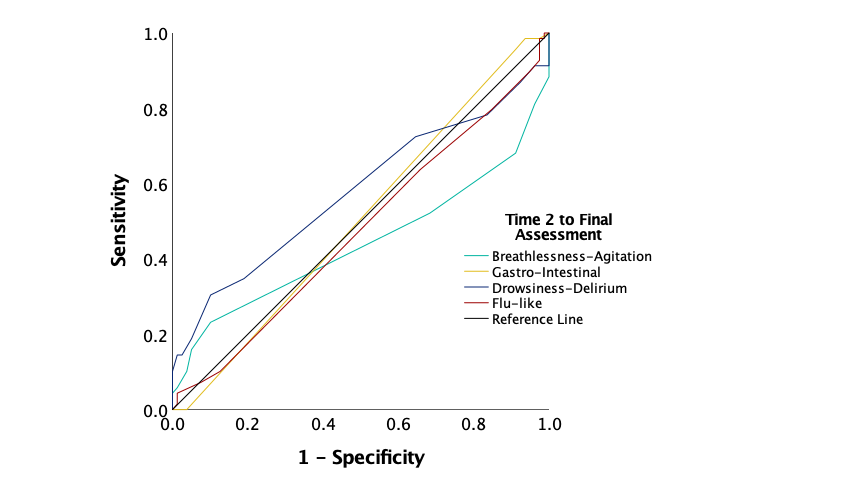
**

Table S 7 Effect sizes in patients who have improved according to PPoI

1. Baseline to time 1 assessment in patient who have improved (n=11)

| **Time interval** | **Subscale** | **Effect size** | **Mean Score in Improved Group** | | **SD in Baseline Stable Group** |
| --- | --- | --- | --- | --- | --- |
|  |  |  | **Baseline** | **Time 1** |  |
| Baseline – Time 1 | BreathAg | 0.3 | 0.8 | 0.4 | 1.5 |
|  | GI | 0.3 | 1.4 | 1.2 | 0.6 |
|  | Drow-Deli | 0.1 | 2.5 | 2.4 | 1.8 |
|  | Flu | -0.1 | 2.5 | 2.7 | 2.6 |

1. Time 1 to time 2 assessment in patient who have improved (n=15)

| **Time interval** | **Subscale** | **Effect size** | **Mean Score in Improved Group** | | **SD in Time 1 Stable Group** |
| --- | --- | --- | --- | --- | --- |
|  |  |  | **Time 1** | **Time 2** |  |
| Time 1 and Time 2 | Breath-Ag | 0.5 | 2.1 | 1.2 | 1.7 |
|  | GI | -0.2 | 0.2 | 0.4 | 1.3 |
|  | Drow-Deli | 0.3 | 3.2 | 2.6 | 1.9 |
|  | Flu | 0.5 | 2.3 | 1.1 | 2.3 |

Table S 8 Minimum Importance Change (MIC) in patients who improved or deteriorated according to CRP levels

|  |  | **Subscale** | **Median Change Score** | **SD** | **n** |
| --- | --- | --- | --- | --- | --- |
| Baseline to Time 1 | Improved | Breath-Ag | 1.0 | 2.2 | 5 |
|  |  | GI | 0.0 | 0.5 | 6 |
|  |  | Drow-Deli | 2.0 | 2.7 | 5 |
|  |  | Flu | 0.0 | 2.2 | 5 |
|  | Deteriorated | Breath-Ag | 0.0 | 1.5 | 3 |
|  |  | GI | 0.0 | 0.0 | 4 |
|  |  | Drow-Deli | 1.0 | 2.5 | 3 |
|  |  | Flu | 1.0 | 2.1 | 5 |
| Time 1 to Time 2 | Improved | Breath-Ag | 1.0 | 4.2 | 2 |
|  |  | GI | 0.0 | 0.0 | 2 |
|  |  | Drow-Deli | 0.5 | 0.7 | 2 |
|  |  | Flu | -2.0 | 1.4 | 2 |
|  | Deteriorated | Breath-Ag | -3.5 | 0.7 | 2 |
|  |  | GI | 0.0 | 0.0 | 2 |
|  |  | Drow-Deli | 2.0 | - | 1 |
|  |  | Flu | 0.0 | - | 1 |
